# BRCA1 testing in Lithuania

**DOI:** 10.1186/1897-4287-10-S4-A9

**Published:** 2012-12-10

**Authors:** Ramunas Janavicius

**Affiliations:** 1 Vilnius University Hospital Santariskiu Clinics Hematology, Oncology and Transfusion Medicine Center, Lithuania

## Background

The germline mutations in BRCA1/2 genes are the most significant and well characterized genetic risk factors for breast and/or ovarian cancer. Detection of mutations in these genes is an effective method of cancer prevention and early detection. Different ethnic and geographical regions may have different BRCA1 and BRCA2 mutation spectrum and prevalence due to founder effect. The population of Lithuania has over several centuries undergone limited mixing with surrounding populations and is mostly of indigenous Baltic origin, which is different from Slavs. The aim of our study was to asses full BRCA1/2 mutational profile in Lithuanian population.

## Methods

We performed comprehensive mutation analysis of BRCA1/2 genes in 605 unrelated breast and/or ovarian cancer patients (with/without family history) and predictive unaffected patients (with family history) using high resolution melting (HRM) screening (Light Cycler 480/Light Scanner 384) followed by direct sequencing (ABI 3500) and MLPA for large genomic rearrangements (LGRs).

## Results

Overall, we have identified 25 different mutations (16 in BRCA1 and 9 in BRCA2 genes). Seven frequent pathogenious mutations in BRCA1 gene (c. 4035delA, c. 5266dupC, c.181T>G, c.1687C>T, c. 5258G>C, c.3700_3704del5, c.4789C>G, del 1-3ex) comprised 48%, 29%, 7,7%, 3,5%, 2,1%, 1,4% and 1,4% respectively of all BRCA1 mutations; a single BRCA2 mutation (c.658delGT) comprised 43% of all mutations in this gene. Five novel BRCA1 (c.4516delG, c.2481delA, c.5560delC, del 16-19ex, del 3-12ex) and 4 novel BRCA2 genes mutations (c.6408_6414del7, c.5701_5714del14, c.6410delA, c.6999insT) were identified; 3 different LGRs (del 1-3ex, del 16-19ex, del 3-12ex) were found in BRCA1. The most common c.4035delA (48% of all BRCA1/2 mutations) appears to be true Lithuanian (Baltic) founder mutation and haplotype data confirmed ancient origin of this mutation c.a. 62 generations ago.

## Conclusions

Characterization of BRCA1/2 mutational profile in Lithuania enabled to develop screening protocol using HRM for 8 common BRCA1/2 point mutations, which comprise 88% of all mutations detected in our country. This knowledge will provide more efficient approach for the individualization of genetic testing affordable for all breast/ovarian patients and their relatives.

